# Expression of Mast Cell Proteases Correlates with Mast Cell Maturation and Angiogenesis during Tumor Progression

**DOI:** 10.1371/journal.pone.0040790

**Published:** 2012-07-18

**Authors:** Devandir Antonio de Souza, Vanina Danuza Toso, Maria Rita de Cássia Campos, Vanessa Soares Lara, Constance Oliver, Maria Célia Jamur

**Affiliations:** 1 Department of Cell and Molecular Biology and Pathogenic Bioagents, Faculdade de Medicina de Ribeirão Preto – University of São Paulo, Ribeirão Preto, São Paolo, Brazil; 2 Department of Estomatology, Faculdade de Odontologia de Bauru, University of São Paulo, São Paolo, Brazil; University of Illinois at Chicago, United States of America

## Abstract

Tumor cells are surrounded by infiltrating inflammatory cells, such as lymphocytes, neutrophils, macrophages, and mast cells. A body of evidence indicates that mast cells are associated with various types of tumors. Although role of mast cells can be directly related to their granule content, their function in angiogenesis and tumor progression remains obscure. This study aims to understand the role of mast cells in these processes. Tumors were chemically induced in BALB/c mice and tumor progression was divided into Phases I, II and III. Phase I tumors exhibited a large number of mast cells, which increased in phase II and remained unchanged in phase III. The expression of mouse mast cell protease (mMCP)-4, mMCP-5, mMCP-6, mMCP-7, and carboxypeptidase A were analyzed at the 3 stages. Our results show that with the exception of mMCP-4 expression of these mast cell chymase (mMCP-5), tryptases (mMCP-6 and 7), and carboxypeptidase A (mMC-CPA) increased during tumor progression. Chymase and tryptase activity increased at all stages of tumor progression whereas the number of mast cells remained constant from phase II to III. The number of new blood vessels increased significantly in phase I, while in phases II and III an enlargement of existing blood vessels occurred. *In vitro,* mMCP-6 and 7 are able to induce vessel formation. The present study suggests that mast cells are involved in induction of angiogenesis in the early stages of tumor development and in modulating blood vessel growth in the later stages of tumor progression.

## Introduction

Mast cells are gaining increased recognition as immunomodulators playing a role in a wide variety of physiological processes [1], [2]. There is increasing evidence that mast cells are associated with various types of tumors such as skin [3], breast [4], lung [5], kidney [6], stomach [7] melanoma [8], and multiple myeloma [9]. Several of these studies correlate mast cell accumulation with angiogenesis, suggesting that mast cells are directly related with blood vessel formation inducing tumor progression [10], [11], [12], [13], [14]. The tumor microenvironment likely facilitates angiogenic responses, resulting in increased blood supply, increased vascular permeability, and extravasation of diverse cytokine-producing cells which may include lymphocytes, macrophages and mast progenitors [15], [16].

One major route by which mast cells could affect various pathways, including angiogenesis is through the effects of mediators such as vascular endothelial growth factor (VEGF), fibroblast growth factor (FGF), IL-8, metalloproteases, serine proteases among others [17], [18], [19], [20] that are stored within the mast cell secretory granules and released upon mast cell activation [1], [21], [22]. However, these mediators are not specific to mast cells and are expressed by other cell types involved in tumor progression. Additionally, previous studies on the role of mast cells in tumorigenesis failed to analyze maturation of mast cell associated with the tumor.

While mature mast cells are easily identified in tissues, immature and very immature mast cells are difficult, if not impossible to identify, due to the scarcity of secretory granules in these cells. Therefore, the number of mast cells and their involvement in tumor progression may be severely underestimated. The maturation of mast cell has been divided in three distinct stages of maturation, very immature, immature and mature. These stages are based on heparin content, granule number and size of the mast cells. Very immature mast cells contain few granules and do not stain with toluidine blue. Immature mast cells have a few small cytoplasmic granules and stain weakly with toluidine blue. In contrast, mature mast cells have a cytoplasm replete with secretory granules and stain strongly with toluidine blue [22], [23], [24]. Because of the difficulty in identifying very immature and mature mast cells, the contribution of mast cells to angiogenesis during tumor progression remains unclear. Hence, the purpose of the present study was to evaluate the role of mast cells during tumor progression. For this purpose tumors were induced by chemical carcinogenesis in BALB/c mice. The recruitment of mast cells to the tumor site as well as their stage of maturation was characterized using mast cell specific antibodies [25], and the expression of tryptase and chymase subtypes and carboxypeptidase A was analyzed during tumor progression. In addition, the relationship between mast cells and neovascularization of the tumor was investigated. The results show that mast cell maturation correlates with tumor progression and angiogenesis in the skin tumor.

## Results

### Skin Tumor Formation

**Figure 1:**
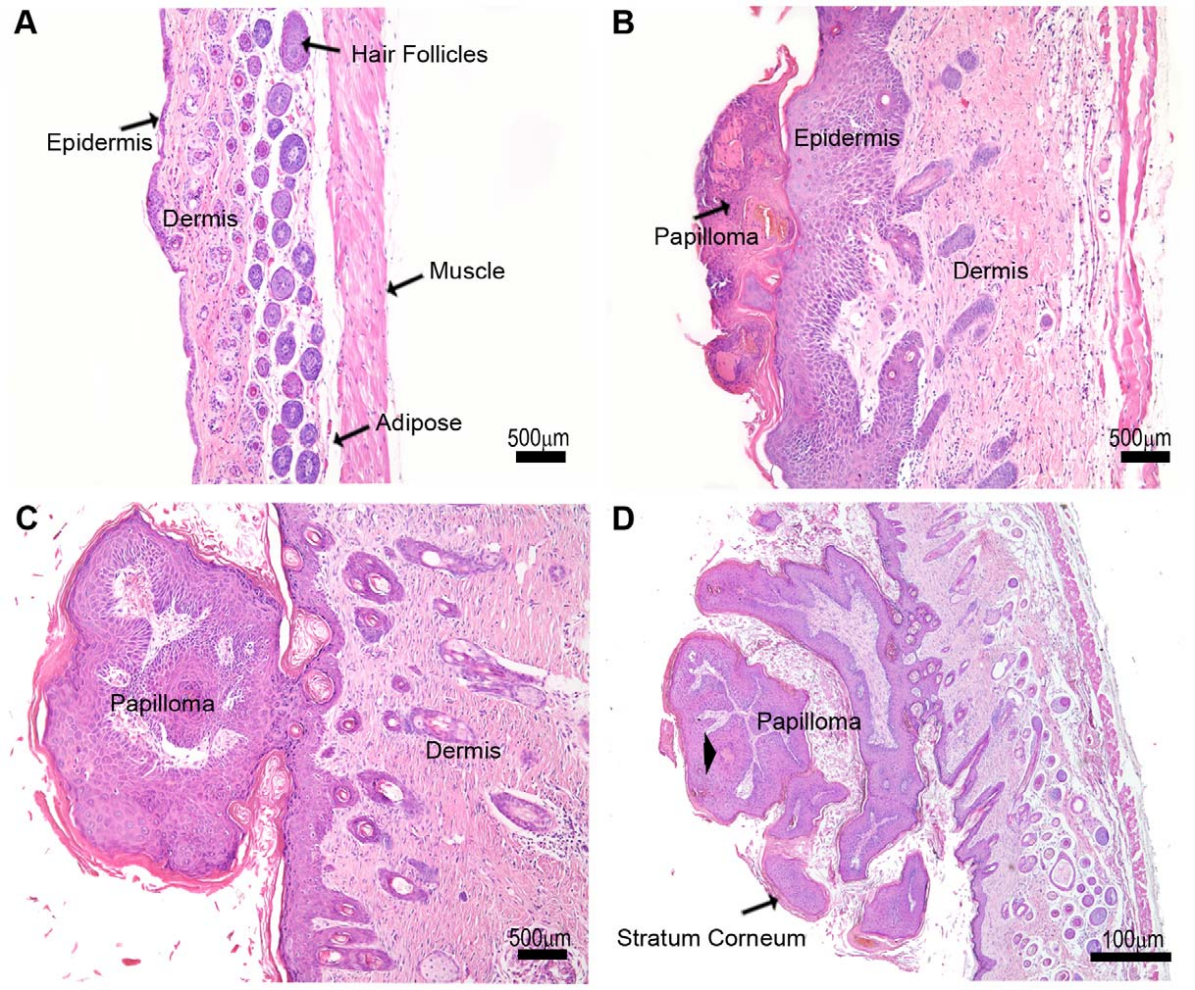
Tumor morphology changes with tumor progression. (**A**) Control –The characteristic organization and thickness of epidermis and dermis are observed in a section of normal skin from a control mouse. (**B**) Phase I –This phase is characterized by epidermal proliferation, a pronounced thickening of the dermis and the formation of a papilloma. (**C**) Phase II - The dermis is thicker and the papilloma is more prominent. (**D**) Phase III - Keratin cysts (*) are observed, as well as a thickening of the stratum corneum.

Local administration of DMBA/TPA induced the formation of skin tumors at 5–12 weeks of administration. Tumor progression was divided into three morphological phases (phase I, II and III) based on the morphological characteristics of the tumor. In the control animals, the skin was comprised of a thin keratinized epidermis overlying the dermis which consisted of fibrous connective tissue with numerous glands and hair follicles. The hypodermis, containing adipose tissue and muscle was below the dermis (Fig. 1A). In Phase I the lesions resembled the early follicular papilloma described by Sundberg *et al*. [26], and were characterized by exophytic interfollicular epidermal proliferation with slight hyperorthokeratosis and a granular layer, consisting of 10 to 15 layers of epithelial cells. The epidermal proliferation included proliferation of the outer root sheath of affected hair follicles and was above the level of the sebaceous glands. A discrete mononuclear inflammatory infiltrate in the superficial dermis was present, sometimes leading to a basal cell infiltration and degeneration. The cutaneous annexes were less numerous than in the control (Fig. 1B). In Phase II the lesions were similar to the exophytic papilloma described by Sundberg *et al.* [26] with an epidermal proliferation raised prominently above the surface of the skin on a narrow stalk. Papillary projections of were composed of 10 to 15 layers of proliferating epithelium and were supported by a thin fibrovascular stroma. The granular layer and hyperorthokeratosis were more evident. The lesions were above the level of the sebaceous glands. Subjacent fibrous connective tissue, were more cellularized than in the previous phase. There was a discrete inflammation around the skin appendages which were less numerous than in the control tissue (Fig. 1C). In Phase III the lesions were similar to those seen in Phase II, but with papillomatosis more pronounced. The papillary projections were covered by 10 to 15 layers of epithelial cells and had a more noticeable granular layer and hyperorthokeratosis, resembling the hyperkeratotic papillomas described by Sundberg *et al.* [26]. Epithelial projections were supported by a fibrovascular stroma that was denser and more cellular than that seen in the previous phase. The lesions were above the level of the sebaceous glands. The subjacent dermis showed less cutaneous annexes than in the control (Fig. 1D).

Tumor progression was further confirmed by evaluating the expression of markers of tumor progression, the integrin subunits αV and β3 [27], [28], [29]. The expression of both the αV and β3 subunits were increased in phase I when compared to controls, remained virtually unchanged in phase II, and increased again in phase III (data not shown).

### The Mast Cell Population Increases During Tumor Progression

**Figure 2:**
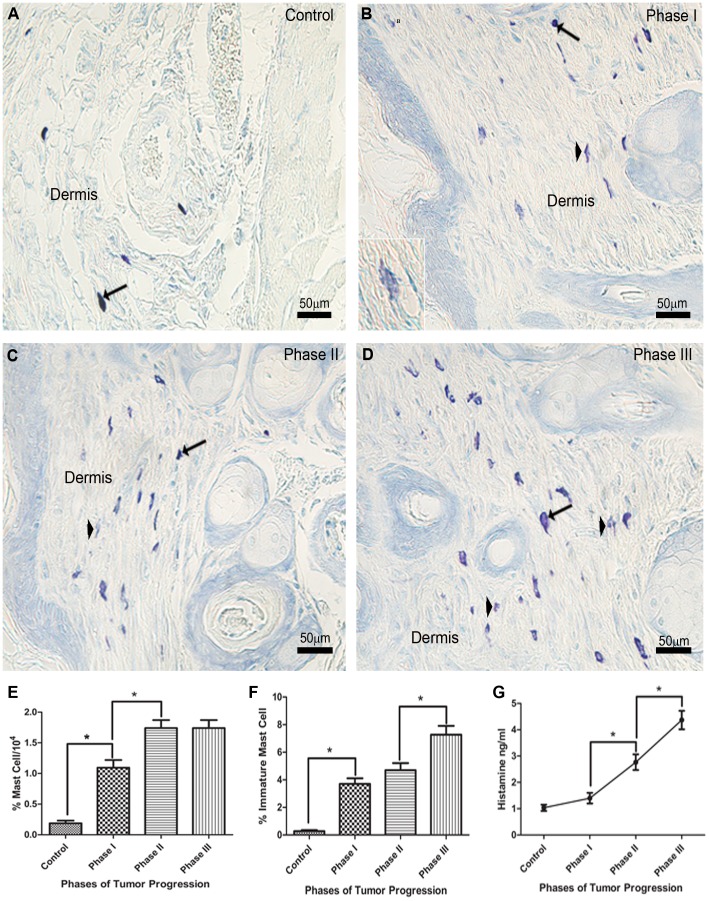
Mature and immature mast cell numbers increase in the dermis during tumor progression. (**A**) Mature metachromatic mast cells (arrow) are observed in the dermis of a control mouse. (**B**) At phase I a pronounced increase in mature (arrows) and immature lightly metachromatic mast cells (arrowhead) are observed in the dermis. In phases II (**C**) and III (**D**) the number of mature (arrows) and immature mast cells (arrowheads) is higher than in phase I. Inset in phase I show an immature mast cell (Toluidine blue). (**E**) Flow cytometry analysis of mast cells during tumor progression using AA4-FITC. (**F**) The percentage of immature mast cells is increased at phase I of tumor progression and continues to increase during the phases II and III. (**G**) Histamine content also increased during tumor progression. (n = 3) *P<0.05.

The mast cell population was evaluated during the three phases of tumorigenesis. In all three stages of tumorigenesis, the stage of maturation of granulated mast cells was determined by staining the sections with toluidine blue and assessing the degree of metachromasia in each mast cell. An increase in the number of metachromatic mast cells in the dermis was observed in all three phases of tumor progression (Fig. 2A–D). In addition, starting at phase I, immature mast cells were observed in the dermis (Fig. 2B inset). Immunostaining with mAb AA4, which recognizes mast cells in all stages of maturation including very immature mast cells that are not metachromatic, was used to identify mast cells by flow cytometry. This analysis showed an approximately 5.5 fold increase in the percent of AA4^+^ mast cells in phase I of tumorigenesis, and an up to 8 fold increase in the percent of AA4^+^ mast cells in phases II and III (Fig. 2E). While there was no increase in the percent of total mast cells in phases II and III, the percent of immature mast cells (Fig. 2F) increased during these phases. The increase in histamine content during phases I-III (Fig. 2G), reflected the increase in immature mast cells seen at these same phases.

### Mast Cell Protease Levels Change During Tumor Progression

**Figure 3:**
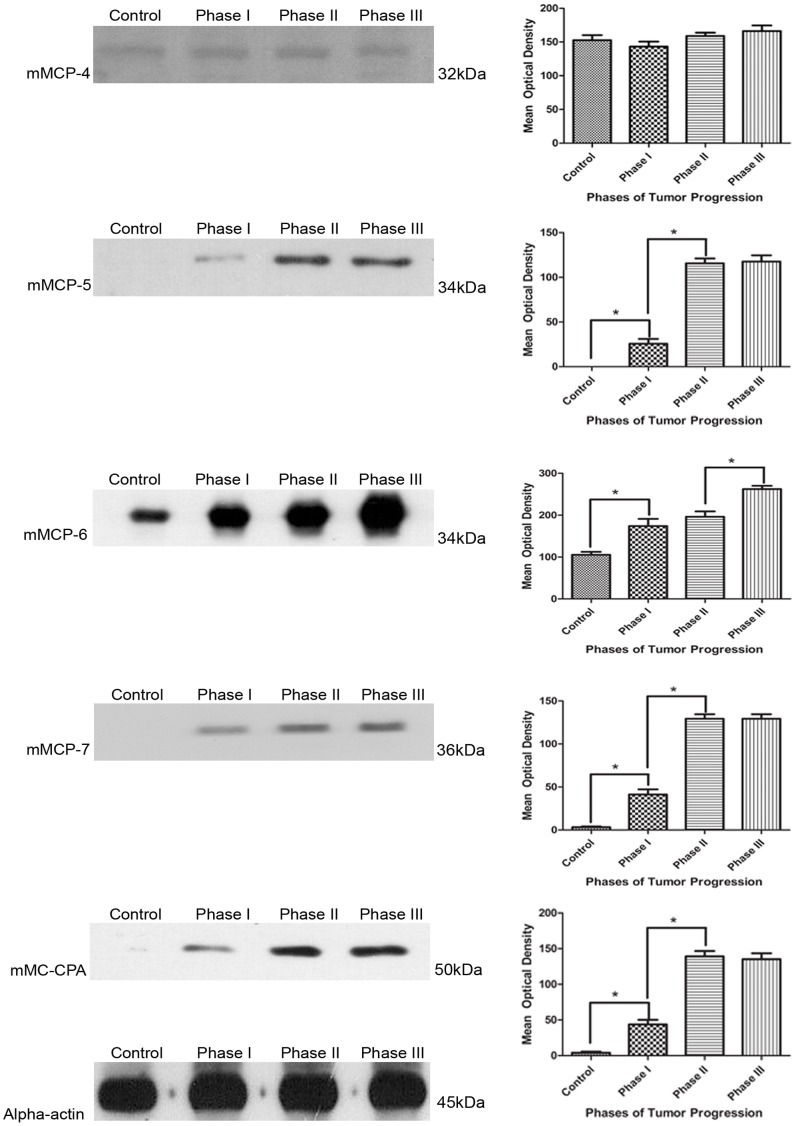
Expression of chymase, tryptase and carboxypeptidase A were altered during tumor progression. Western blots of cell lysate from control, phases I, II and III of tumor progression. The levels of mMCP-4 are constant in the 3 phases. mMCP-5 is only expressed during tumorigenesis. The levels of mMCP-6 increase progressively during the three phases. mMCP-7 was not detected in control animals, begging to expression in phase I and remained unchanged in phases II and III. The expression of mMC-CPA is almost not detectable in controls, but increases in phases I and II, and remains unchanged in phase III. Alpha-actin was used as a loading control. Mean optical density of blots presenting the data of the mean values ± SEM of 3 independent experiments. *P<0.05.

Although there was no difference in the number of AA4^+^ mast cells during phases II and III, the histamine content increased. Therefore, it was of interest to determine if there was an increase in the expression of chymase, tryptase or CPA during tumor progression mainly in phases II and III. The levels of mast cell granule specific proteases, chymases (mMCP-4 and mMCP-5), tryptases (mMCP-6 and mMCP-7) and mMC-CPA were analyzed (Fig. 3). mMCP-4 was expressed in control animals and in all three phases of tumorigenesis, but did not show differences in expression at any phase. In contrast, mMCP-5 was expressed only during tumorigenesis. mMCP-6 levels increased approximately two fold during tumor progression when compared to the control. mMCP-7 was not detected in control animals, but is expressed in phase I and its expression increased during the late phases (II and III). mMC-CPA had a very low level of expression in control animals, but it increased with tumor progression. During phases II and III, there was an increase in the expression of chymase, tryptase, and CPA, but this was not accompanied by an increase in the number of AA4^+^ mast cells.

### Enzymatic Activities of Chymase and Tryptase Increase During Tumor Progression

**Figure 4:**
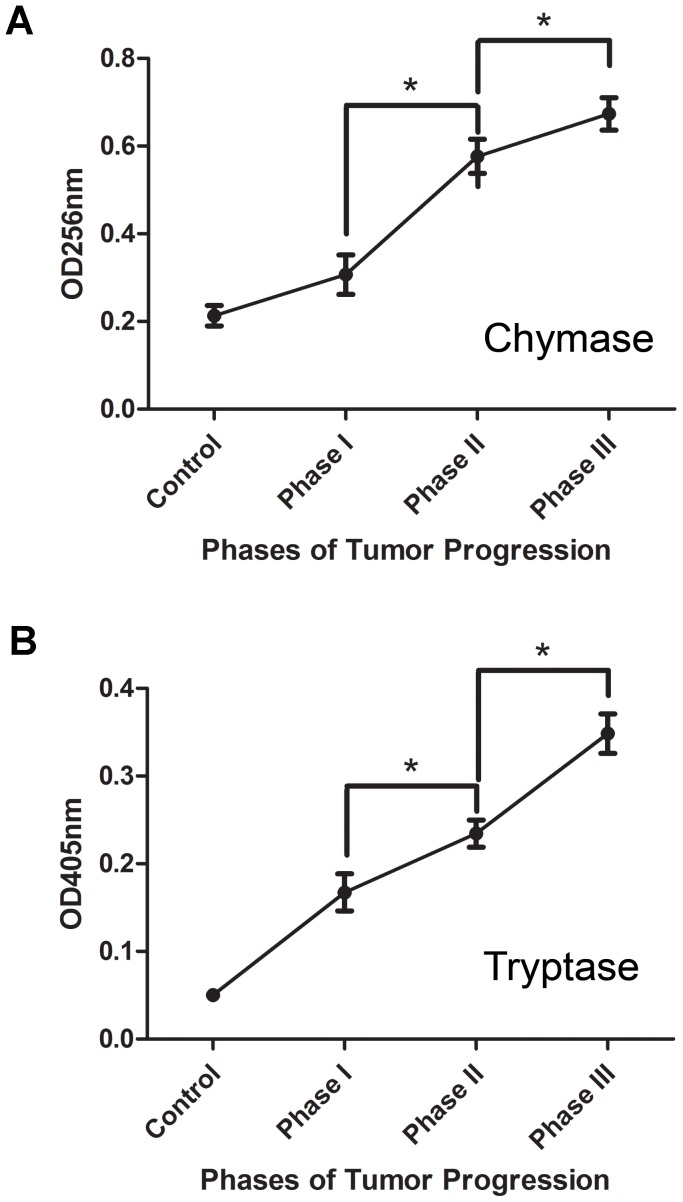
Enzymatic activity of chymase and tryptase increase with tumor progression. The activities of both chymase and tryptase increase mainly in phases II and III. The data represent the mean values ± SEM. (n = 3) *P<0.05.

The increase in the levels of expression of chymase and tryptase correlated with their enzymatic activities which increased about 4 fold during tumor progression (Fig. 4). Chymase and tryptase activities increased progressively from phase I through phase III of tumorigenesis. This increase in mast cell protease activity suggests a direct involvement of mast cells in tumor progression.

### Blood Vessel Number and Diameter Increase During Tumor Progression

**Figure 5:**
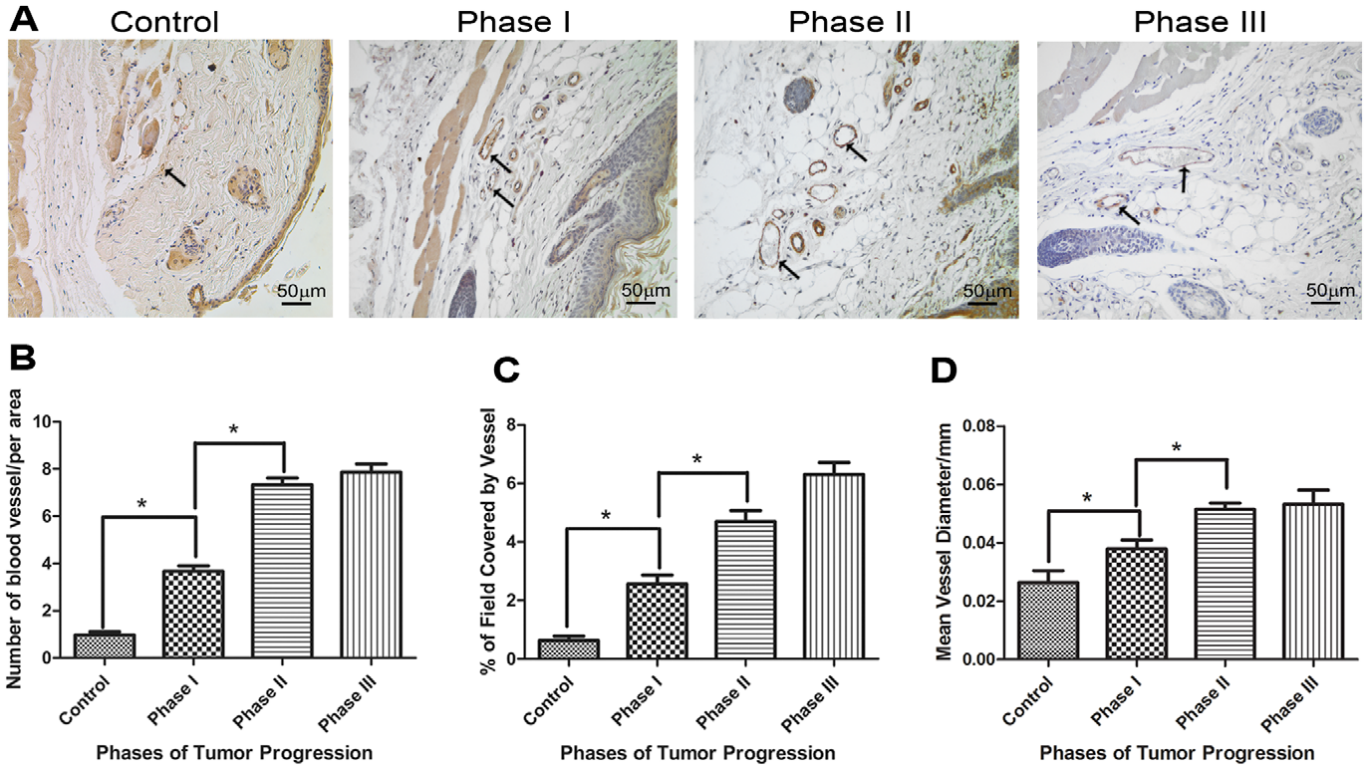
Blood vessel number and diameter increase during tumor progression. (**A**) Representative sections of control skin and tumors during Phases I, II and III demonstrating the increase in the number and diameter of blood vessels (arrows) with tumor progression. Blood vessels were immunostained for von Willebrand factor. (**B**) The number of blood vessels, (**C**) the percentage of area covered by blood vessels and (**D**) the diameter of the vessels increases considerably in phase I and II and remains constant in phase III. (n = 3) *P<0.05.

To correlate blood vessel formation during tumor progression, blood vessels were immunostained for von Willebrand factor and analyzed in histological sections (Fig. 5A). An increase in the number of blood vessels occurred in phase I, further increased in phase II, and remained constant in phase III (Fig. 5B). In parallel with the increase in blood vessel number, there was also an increase in the area occupied by blood vessels beginning in phase I that continued through phases II and III (Fig. 5C). Additionally the diameters of vessels were quantified and the results show that their diameter increases in phase I and II and remains unchanged at later phases (Figure 5D).

### Tryptase Accelerates Angiogenesis in vitro

**Figure 6:**
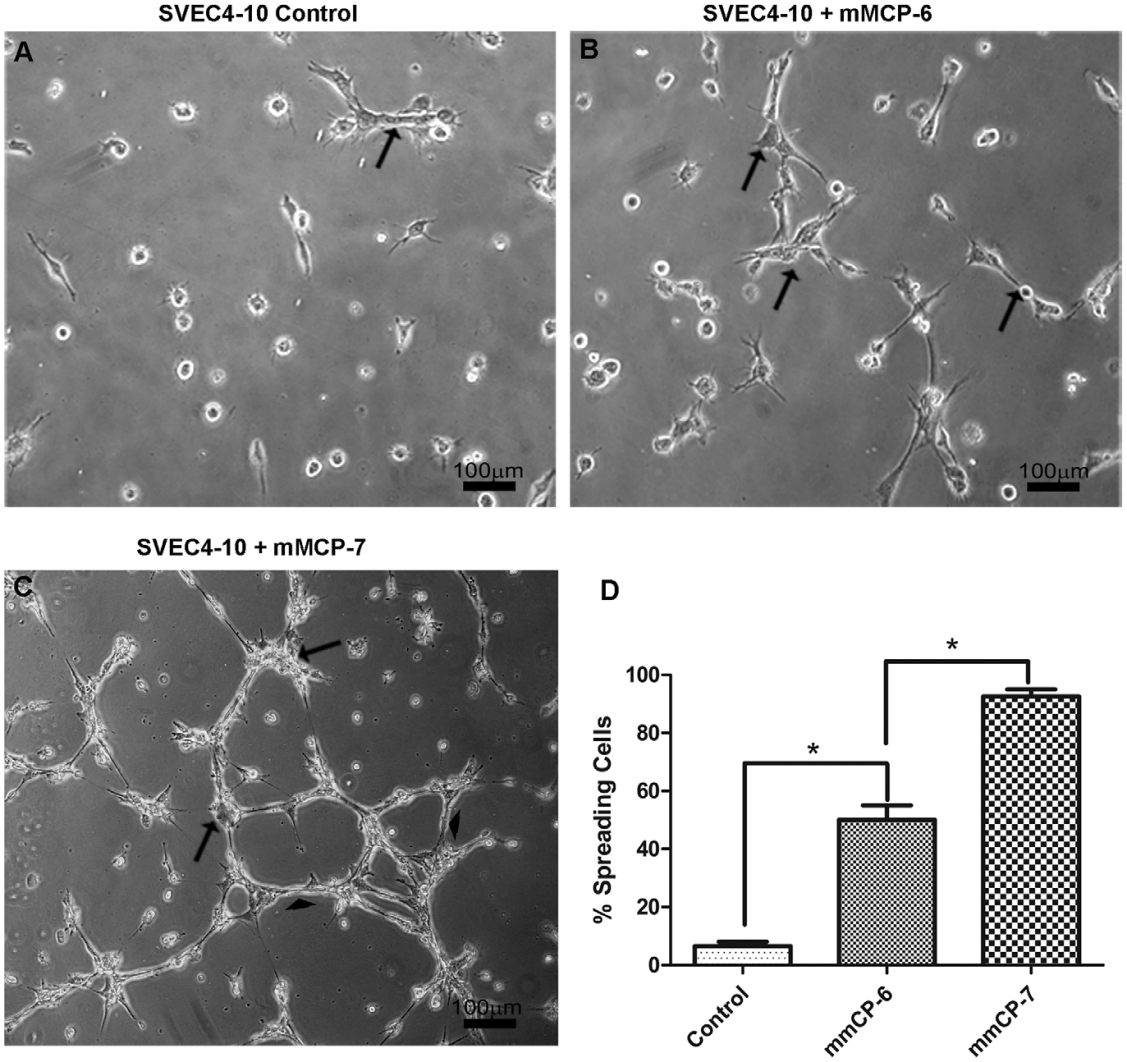
Mast cell proteases induce cell spreading and tube formation in SVEC4-10 cells. (**A**) When cells were incubated in absence of proteases, only a few cells were spread on the surface, beginning to form tubes (arrow) and most of the cells remained unspread. (**B**) mMCP-6 augments cell spreading and tube formation in comparison to the control cultures (arrows). (**C**) mMCP-7 is more efficient in inducing cell spreading and tube formation than mMCP-6. Most of cells are spread (arrowhead) or in the form of tubes (arrows). Phase contrast microscopy. (**D**) The graphics show the percent of cells that were spread on the surface (Geltrex™) after 5 h incubation. *P<0.05.

To directly evaluate the ability of specific mast cell tryptases to induce angiogenesis, a tube formation assay was performed using the mouse epithelial cell line SVEC4-10. The capacity of mast cell tryptases, mMCP-6 and mMCP-7, to increase cell spreading and tube formation was evaluated (Fig. 6). In control cultures with no proteases, the SVEC4-10 cells only 5.2% ±3,1% were spread on the surface (Fig. 6A). When cells were cultured for 5 h in the presence of mMCP-6, 45.4% ±9.8% of the cells were spread and formed endothelial tubes. In the presence of mMCP-7, 90.5% ±5.2% of the cells were spread or in the form of tubes (Fig. 6C). mMCP-7 induced cell spreading and tube formation to a greater extent than mMCP-6 (Fig. 6D).

## Discussion

The present study shows that the expression of mast cell chymase and tryptase subtypes as well as carboxypeptidase A varies during tumor progression. Furthermore, we observed that increased expression of these proteases correlated with mast cell maturation and blood vessel formation, indicating their direct involvement in skin tumor progression.

The changes in the mast cells population observed during tumor progression suggests that total number of mast cells (mature plus immature) increases during phase I and II and, remains stable through phase III. The accumulation of mast cells in tumors may be a response to the various chemoattractant factors produced by mast cells and other cell types associated with the tumors [30]. *In vitro* studies have shown that certain angiogenic factors such as VEGF, PDGF, and bFGF are also chemoattractants for mast cells [31]. Although several studies have shown that mast cells accumulate around tumors [32], [33], there is no information about changes in the mast cell population or their maturation during tumor progression. Immature mast cells initially appear at the beginning of tumor formation and their number increases with tumor progression. However, the increase in mediator content, particularly in the late phases, is likely to be related to mast cell maturation.

The role of mast cells during tumor progression can be directly related to their granule content. Our results revealed changes in the expression of some mast cell-specific chymases, tryptase and carboxypeptidase A during tumor progression. Initial expression of mMCP-5 correlated with the initiation of tumorigenesis and its expression level increased in the later phases of tumor progression. This increase in mMCP-5 expression may be related to mast cell maturation. mMCP-5 is known to be one of the first mast cell proteases expressed during maturation [34]. The results of the present study suggest that in phase I most mast cells at the tumor site are either very immature or immature cells that express low levels of mMCP-5.

mMC-CPA expression also increased significantly with the appearance of the tumor and during tumor progression. Until recently, little information about the *in vivo* function of mMC-CPA was available. Recent evidence suggests that the storage of mMC-CPA and mMCP-5 in mast cell granules are interdependent. mMC-CPA knockout mice, lack mMCP-5 [35], and conversely, mice deficient in mMCP-5 lack mMC-CPA but not mMC-CPA mRNA [36], [37]. A possible explanation for the interdependence between mMC-CPA and mMCP-5 is that they bind to each other forming a protein complex [38] which may have functional implications. The close association of the two proteases in the secretory granule would facilitate substrate degradation upon release [38]. Our results show that expression of mMC-CPA and mMCP-5 follow a similar pattern during tumor progression and agree with other studies in different model systems [39], [40]. Lundesquist and collegues [19] have shown that chymase and mMC-CPA cooperate in the processing of angiotensin I, thus these protease complexes could be critical for neovascularization during tumor progression.

mMCP-6 expression increases dramatically during tumor progression, suggesting that this protease also has a role in the early and late stages of tumor progression. In contrast, mMCP-7 was only detected during tumor progression, suggesting these proteases have a pivotal role in tumor progression. Several studies have related mast cell tryptases to enhancement of vascular permeability [41], tissue remodeling [42] and recruitment of inflammatory cells to a site of inflammation [43]. mMCP-6 and mMCP-7 are functionally distinct tryptases [37], [44], [45]. Therefore, these tryptase subtypes may be acting differentially during tumor progression. Recent studies show that mMCP-6 induces neutrophil influx while mMCP-7 induces eosinophil influx during infection [46]. These tryptases may also participate in the degradation of extracellular matrix components, an event essential for neovascularization and tumor progression [47], [48].

The relationship between mast cells and tumor progression is unclear; nevertheless, studies on tumor-associated mast cells suggest that these cells participate in tumor progression by promoting local angiogenesis through mediator production [10], [11], [12]. The *in vitro* results from the current study show that mMCP-6 and mMCP-7 are able to induce tube formation in endothelial cells. These data suggest that both subtypes of tryptases released from mast cells associated with the tumors may have a direct role in neovascularization during tumor progression.

The present study shows that the expression of mast cell specific proteases correlates with tumor progression. Furthermore, it appears that immature mast cells are recruited to the tumor site during the initial stages of tumor induction and mature with tumor progression. Additionally, mast cells proteases participate in blood vessel formation and modulation during tumor progression. They are involved in the induction of angiogenesis at the early stages of tumor formation and in modulating blood vessel growth in the later stages. The results of this study provide a better understanding of role of mast cell specific protease in skin tumor progression and may lead to new therapeutic targets.

## Materials and Methods

### Ethics Statement

The research was conducted in accordance with “Ethical principles in the use of experimental animals” adopted by the Brazilian College of Animal Experimentation. Experimental protocols were approved by the Commission on Ethics on Animal Experimentation of the Faculdade de Medicina de Ribeirão Preto (Protocol number 033/2007). Animals were anesthetized with ketamine 80 mg/kg plus xylazine 12 mg/kg (Sigma-Aldrich, St. Louis, MO) prior to experimental treatment.

### Animals

Male BALB/c mice, 4–6 weeks old were used in this study. Animals were housed and experiments were approved and conducted according to FMRP-USP, Ribeirão Preto, Brazil guidelines. Animal experiments were done in triplicate.

### Tumor Production

One topical application of 100 µg of DMBA (7,12-dimethylbenz[a]anthracene) (Sigma- Aldrich, St Louis MO) was used to induce chemical tumorigenesis. One week post-induction animals were treated topically on the dorsal region with 100µl of acetone containing 20 µg of TPA (12-O-tetradecanoilforbitol) (Sigma-Aldrich) [49]. The TPA was further applied twice a week until the tumor achieved the desired phase. Controls received acetone only.

### Antibodies

mAb AA4 recognizes mast cell specific α-galactosyl derivatives of the ganglioside GD1b (GUO *et al*. 1989; OLIVER *et al*. 1992). Rabbit anti-mMCP-4, rabbit anti-mMCP-5, and rabbit anti-mMCP-6 were kindly provided by Dr. Michael F. Gurish (Division of Immunology, Children’s Hospital, Harvard Medical School, Cambridge, MA). Rabbit anti-von Willebrand factor was purchased from Millipore (Billerica, MA). Rabbit anti-integrin αV, rabbit anti-integrin β3, goat anti-mouse mMCP-7, rat anti-mouse mMC-CPA and rabbit anti-alpha actin were purchased from Santa Cruz Biotechnology (Santa Cruz, CA).The secondary antibodies goat anti-rabbit HRP and donkey anti-rat HRP were purchased from JacksonImmunoResearch (West Grove, PA). Controls consisted of omitting the primary antibody or substituting the primary antibody directly conjugated to FITC with normal IgG from the same species conjugated to FITC.

### Microscopy

Tumors were removed using a 13 mm punch. Excised tumors were washed in PBS and fixed in 4% formaldehyde (EM Sciences, Hatfield, PA) in PBS. The tumors were processed for routine histology, stained with either hematoxylin and eosin or toluidine blue (0.1%, pH2.8), and mounted on glass slides with Permount (Thermo Fisher Scientific Inc., Waltham, MA). Some slides were used for immunohistochemistry. Briefly, sections mounted on glass slides were deparaffinized and rehydrated, and endogenous peroxidase activity was blocked with 3% H_2_0_2_ in methanol. Non-specific binding was blocked with PBS containing 2% BSA for 45 minutes and then samples were incubated with the primary rabbit anti-human von Willebrand factor (10 µg/ml) for 1 h at room temperature (RT). Following incubation, the sections were rinsed thoroughly in PBS and the samples incubated with anti-rabbit IgG conjugated to HRP (Jackson ImmunoResearch) for 45 minutes. After which, the slides were rinsed thoroughly in PBS and incubated with 3,3′-diaminobenzidine and H_2_0_2_ for 15 minutes, mounted with Permount. Samples were observed using an Olympus BX-50 microscope (Olympus America Inc., Melville, NY) equipped with a Nikon DXM-1200 digital camera (Nikon USA, Melville, NY).

### Tumor Dissociation

The tumors were dissociated according to the method of Bradshaw [50], with modifications. Briefly, the tumors were harvested and placed into sterile 6 wells plates containing 0.6 units/ml of trypsin (Worthington Biochemical Corp, Lakewood, NJ) in dissection buffer (10 mM glucose, 3 mMKCl, 130 mMNaCl, 1 mM Na_2_HPO_4_, 30 mM HEPES, pH 7.4) and incubated overnight at 4°C. After incubation the epidermis was separated from the dermis. The dermis underwent further digestion with 750units/ml of type IV collagenase (Worthington) in DMEM for 3 h at 37°C. The cells were filtered through 25 µm nylon mesh and the cell suspension was centrifuged at 240 *g* at 4°C for 10 minutes. The supernatant was discarded; the pellet resuspended in PBS and the cells used for flow cytometry.

### Flow Cytometry

After dissociation cells were fixed with 2% formaldehyde (EM Sciences) blocked with PBS containing 1% BSA (Sigma-Aldrich) for 45 minutes and incubated with mAb AA4 (5 µg/ml) for 1h conjugated to FITC (Jackson ImmunoResearch). Following incubation, cells were washed by centrifugation at 240 *g* in PBS and analyzed by flow cytometry using a Becton-Dickinson FACSCalibur™ (Becton-Dickinson Biosciences; Bedford, MA) with CELLQuest software.

### Tumor Lysate

Tumor lysates were prepared using a modification of the ProSciIncoporated Protocol (Poway, CA). Tumors were removed using a 13 mm punch and then homogenized in lysis buffer (10 mM HEPES pH7.9, 1.5 mM MgCl_2_, 10 mM KCl, 1 mM EDTA, 10% glycerol, 1% NP-40, and 15 µl of protease inhibitor cocktail [Sigma-Aldrich]) using an Ultra80 Homogenizer (Ultra Stirrer, Eikonal do Brasil, São Paulo, SP). Tissue and cell debris were removed from the lysate by centrifugation (2 800 *g*/4°C/30minutes). The protein concentration of the supernatant was determinate using a BCA Protein Assay Kit (Pierce, Thermo Fisher Scientific, Rockford, IL).

### Immunoblotting

20 µg of lysate were boiled for 5 minutes in 1× SDS sample buffer (50 mM Tris-HCl pH 6.8, 12.5% glycerol, 1% sodium dodecylsulfate, 0.01% bromophenol blue), and applied to10% polyacrylamide gels. Proteins were then separated electrophoretically and transferred to Hybond membranes (GE Healthcare, Piscataway, NJ). Membranes were blocked overnight in TBS (0.05 M Tris–HCl, 0.15 M NaCl, pH 7.5, and 0.05% Tween 20) containing 5% nonfat dry milk at 4°C. Membranes were then incubated with anti-protease antibodies (mMCP-4, mMCP-5, mMCP-6, mMCP-7 and mMC-CPA) at 1∶200 or rabbit anti-integrin αV, rabbit anti-integrin β3, for 1 h at RT, washed in TBS/Tween and incubated with goat anti-rabbit IgG conjugated to HRP or goat anti-rat IgG conjugated to HRP (1∶20,000) (JacksonImmunoResearch) for 30 minutes at RT, washed and developed using chemiluminescence (ECL-GE Healthcare, Piscataway, NJ). Alpha-actin was used as a loading control. The blots were scanned, and the optical densities of the bands were calculated using Adobe Photoshop (Adobe Systems, San Jose, CA).

### Histamine Assay

Histamine assays were performed as previously described [51]. Briefly, the histamine was extracted into n-butanol from alkalinized perchloric acid tissue extracts. The histamine was returned to an aqueous solution and condensed with o-phthalaldehyde (Sigma-Aldrich) to yield a fluorescent product. The intensity of fluorescence was measured using the Histamine Module for the Trilogy Laboratory Fluorometer (Turner Designs, Inc., Sunnyvale, CA).

### Chymase and Tryptase Activity

Chymase activity was determined using the Chymase Assay Kit (Sigma-Aldrich) using the mast cell specific substrate the N-benzol-L-Tyrosine ethyl ester (BTEE) and read at 256 nm. Tryptase activity was determined using the Mast Cell Degranulation Assay Kit (Millipore) with the mast cell specific substrate the tosyl-gly-pro-lys-*p*NA with substrate and measured at 405 nm. For these assays, lysates were prepared at 4°C without protease inhibitors and stored at −80°C until use. There was no difference in mg protein/ml between lysates prepared with or without protease inhibitors. Both assays were evaluated using an Elisa Power Wave X Plate Reader (Bio-Tek Instruments, Inc., Winooski, VT).

### Blood Vessel and Mast Cell Quantification

Vessel counting was performed as described by Weidner [52]. After immunostaining with von Willebrand factor, each slide was screened using a 20× objective to identify the center of the tumor. An area of 1,9 mm^2^ was delineated using the center of the tumor as a marker. The total number of vessels, their area and diameter were determined in a delineated region for three slides per animal. The vessel count for each animal was expressed as the average number of vessels from five different slides. The area covered by vessels as well as their diameter was determined in the same fields screened for vessel counts. The percentage of the area covered by vessels in each animal was expressed as the mean from the five slides analyzed. The area of each blood vessel was determined using Image-Pro Plus, v 5.5.1.38 (MediaCybernetics, Bethesda, MD). Mast cells were counted after staining sections with toluidine blue. The same procedure was used to quantify toluidine blue mast cells as was used for blood vessels. This procedure was used for quantifying randomly selected areas of the dorsal derme of control animals.

### In vitro Assays

The murine endothelial cell line SVEC4-10 was purchased from the American Type Culture Colletion (Manassas, VA). The cells were maintained in DMEM (Invitrogen Corporation, Carlsbad, CA) plus 10% fetal bovine serum in a humidified environment containing 5% CO_2_ in air. Geltrex™ (Invitrogen) was added to the wells (200 µl/well) of 24 well plates and allowed to solidify at 37°C for 30 minutes, then 4×10^4^ cells in 200 µl of DMEM were placed in each well and incubated at 37°C for 5 h with or without 20 ng of tryptase (mMCP-6 or mMCP-7, R&D Systems Inc., Minneapolis, MN). SVEC4-10 cells spontaneously spread and undergo tube formation. The ability of the cells to form endothelial tubes in the presence or absence of tryptases were evaluated by phase contrast microscopy using a Nikon Eclipse TS100 inverted microscope equipped with a Nikon DXM-1200 digital camera (Nikon).

### Statistical Analysis

Values are expressed as the mean ± SD. Student’s *t*-test was used to compare tumor phases. *P*-values of <0.05 were considered significant. The data are means from three independent experiments.
